# Assessment of Foveal Avascular Zone and Macular Vascular Plexus Density in Children With Unilateral Amblyopia: A Systemic Review and Meta-Analysis

**DOI:** 10.3389/fped.2021.620565

**Published:** 2021-05-21

**Authors:** Lei Gao, Yang Gao, Fengrong Hong, Peng Zhang, Xiangwen Shu

**Affiliations:** ^1^Department of Ophthalmology, Jinan 2nd People's Hospital, Jinan, China; ^2^Department of Ophthalmology, Linqing People's Hospital, Linqing, China

**Keywords:** fovea, macula, angiography, amblyopia, unilateral, meta-analysis, tomography, optical coherence

## Abstract

**Purpose:** To examine the quantitative measurements of OCTA in children with amblyopia using the meta-analysis methodology.

**Methods:** PubMed, Embase, and Cochrane library were searched for available papers up to March 2021. Weighted mean differences (WMD) were used to compare the retina parameters between the eyes with amblyopia and the contralateral eyes or healthy control eyes.

**Results:** Twelve studies were included. When considering the parafovea, the microvessel density was reduced in amblyopic eyes compared with healthy control eyes in the superficial capillary plexus (SCP) in 6 × 6 volume scan (WMD = −2.12, 95%CI: −3.24, −0.99) but not SCP in 3 × 3 volume scan (WMD = −1.43, 95%CI: −2.96, 0.11). In the deep capillary plexus (DCP), amblyopia did not decrease vessel density in the 6 × 6 volume scan (WMD = −2.22, 95%CI: −5.86, 1.42; *I*^2^ = 79.6%, *P* = 0.008), but a difference was observed in the whole eye 3 × 3 (WMD = −1.95, 95%CI: −3.23, −0.67; *I*^2^ = 27.5%, *P* = 0.252). There were no significant differences in the foveal avascular zone area and foveal thickness between amblyopic eyes and healthy control eyes. There were no significant differences in microvessel density, foveal avascular zone area, and foveal thickness between amblyopic eyes and fellow eyes.

**Conclusion:** According to OCTA, amblyopic eyes had lower vessel density in parafoveal SCP and DCP compared with healthy control eyes, but not compared with fellow eyes. There were no significant differences regarding the foveal avascular zone area and foveal thickness between amblyopic and non-amblyopic eyes.

## Introduction

Amblyopia is defined as a reduction in best-corrected visual acuity (2-line difference between the two eyes) secondary to neurological deficits in visual output caused by abnormal brain stimulation during critical periods of visual development (1–3). It is usually unilateral and is the most common cause of vision loss and mononuclear blindness in children. It remains the most common cause of preventable vision loss in children (4). The worldwide prevalence is 1–5%, and the prevalence in North America is 2–4% (2, 3).

In animal models of amblyopia, fewer ganglion cells project to the central visual nuclei (5), and it has been suggested that the postnatal process of ganglion cell reduction is affected by amblyopia and should result in a thicker circumpapillary retinal nerve fiber layer (6). A meta-analysis of optical coherence tomography (OCT) studies revealed that a thicker foveola was found in amblyopic eyes compared with normal eyes (7). On the other hand, it is unknown whether this thicker fovea is associated with changes in blood flow or microvessel density.

OCT angiography (OCTA) is a relatively new and non-invasive imaging technique that uses motion control contrast imaging to obtain high-resolution volumetric blood flow information and generate angiographic images of the retina and choroid (8). The advantages of OCTA over the traditional fluorescein and indocyanine-green angiography are its fast acquisition time and non-invasiveness (9, 10). In addition, it provides quantitative measurements (11). It can be used to assess retinal diseases, glaucoma, and uveitis (9, 10). Importantly, OCTA can provide quantitative assessments of the superficial capillary plexus (SCP) and deep capillary plexus (DCP) (12). Some studies examined the use of OCTA for amblyopic eyes, but the results are inconsistent, and no meta-analysis is available to synthesize the quantitative measurements of OCTA in children with amblyopia.

Therefore, the aim of the present meta-analysis is to examine the quantitative measurements of OCTA in children with amblyopia. The results could provide a firmer basis for the use of OCTA for the management of amblyopia.

## Methods

### Study Selection

This meta-analysis was conducted according to the Preferred Reporting Items for Systematic Reviews and Meta-Analyses (PRISMA) guidelines. The study eligibility criteria were: (1) children with amblyopia in one eye as the case group, compared with the contralateral eyes or healthy control eyes; (2) quantitative measurement of the foveal avascular zone and macular vascular plexus density using OCTA; (3) language limited to English; and (4) the outcomes could be extracted.

PubMed, Embase, and Cochrane library were searched for available papers up to March 2021 using the MeSH terms and key words pertinent to “amblyopia” and “optical coherence tomography angiography.”

### Data Extraction and Quality Assessment

The selection and inclusion of studies were performed in two stages by two independent reviewers (Lei Gao and Xiangwen Shu). This included, first, the analysis of the titles and abstracts, followed by the full texts. Any disagreements were resolved by a third reviewer (Peng Zhang).

Data, including names of authors, publication year, study design, sample size, type of amblyopia, OCTA algorithm, and spherical equivalent, were gathered with a structured data collecting form. The observational studies were evaluated according to the Newcastle-Ottawa scale (NOS) (13, 14).

### Statistical Analysis

All analyses were performed using STATA SE 14.0 (StataCorp, College Station, Texas, USA). Weighted mean differences (WMD) and corresponding 95% confidence intervals (CIs) were used to compare the retina parameters between the eyes with amblyopia and the contralateral eyes or healthy control eyes. Statistical heterogeneity among these studies was calculated using Cochran's *Q*-test and the *I*^2^ index. *Q*-test with *P* < 0.10 and *I*^2^ > 50% indicated high heterogeneity, and the random-effects model was then used; otherwise, the fixed-effects model was used. The publication bias was analyzed using funnel plots. *P*-values < 0.05 were considered statistically significant.

## Results

### Search Process

**Figure 1:**
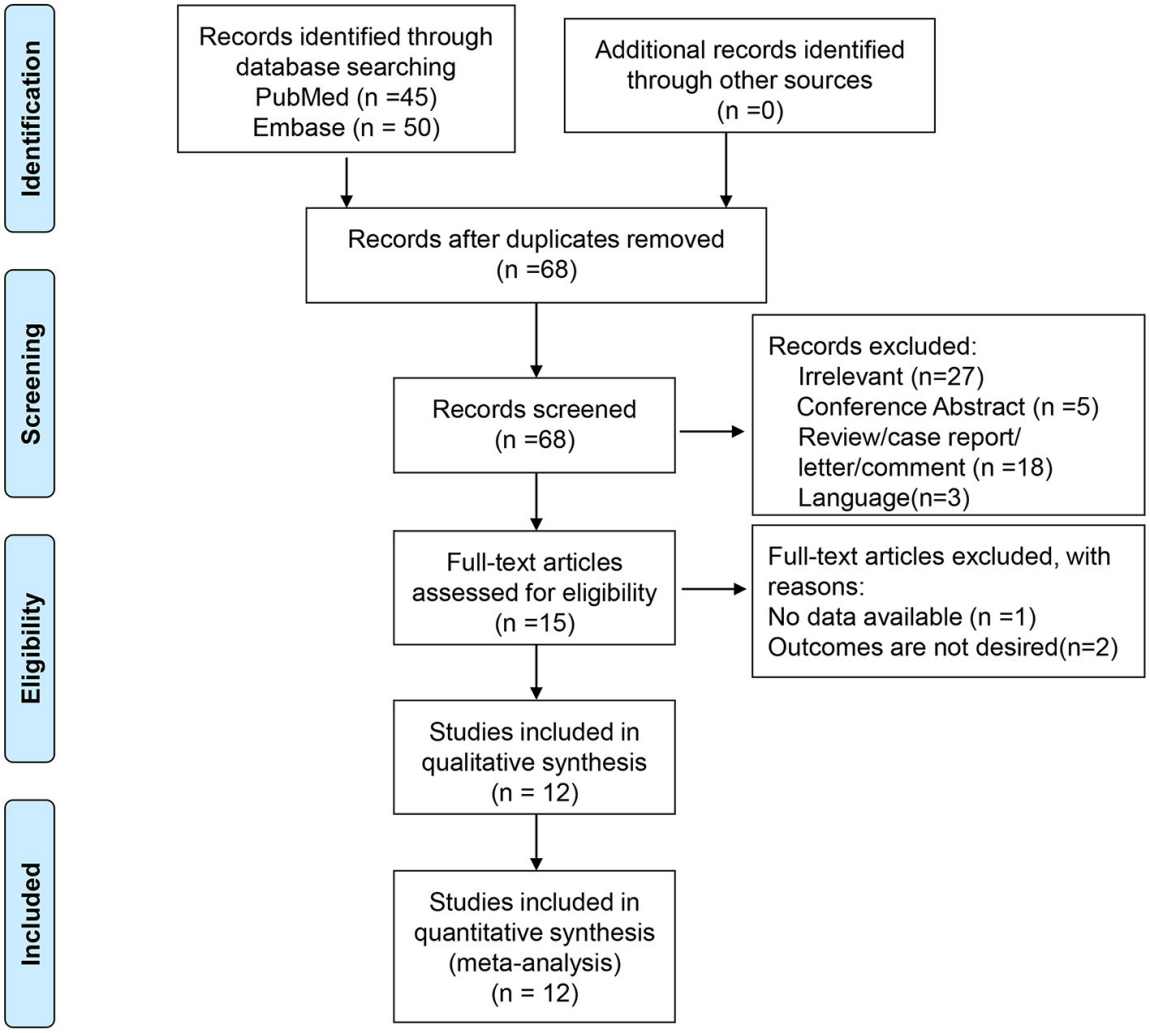
Flowchart of study selection.

A total of 95 entries were retrieved from PubMed and Embase. After removing duplicates, 68 titles and abstracts were screened. After excluding the irrelevant papers and invalid paper types, 15 full-text papers were reviewed. One was excluded for no available data and two for inappropriate outcomes. Therefore, 12 studies were included in the present meta-analysis (15–26) (Figure 1 and Table 1). There were eight cross-sectional studies (15, 16, 18–20, 23, 25, 26), one longitudinal study (17), one cohort study (24), one case-control study (22), and one undefined (21). A total of 1,744 patients were included (range, 12–1,075/study). Table 2 presents the quality of the studies according to the NOS. All 12 studies were of high quality.

**Table 1:** The included studies.

**References**	**Design**	**Sample size**	**Scan size**	**Age (years, mean ± SD)**	**Sex female (%)**	**OCTA** ** algorithm**	**Spherical equivalent (*D*)**	**Type of amblyopia**	**Study location**	**No. of eyes intervention comparator**	
Zhang et al. (26)	Cross-sectional	59	6 × 6 mm	7.86 ± 1.88	41.0%	RTVue XR Avanti (SD)	4.97 ± 2.58	A	China	22	21
Chen et al. (17)	Longitudinal study	88	3 × 3 mm	8 (7, 9)	59.1%	RTVue XR Avanti (SD)	4.44 ± 1.89	A	China	22	66
Cinar et al. (18)	Cross-sectional	74	3 × 3 mm	12 ± 4.2/13 ± 6.1	51.4%	Triton DRI-OCT	3.80 ± 2.20	A	Turkey	37	37
Gunzenhauser et al. (21)	/	12	3 × 3 mm	6.5	66.7%	RTVue XR Avanti (SD)	/	Mixed (A, AS)	USA	12	/
Wong et al. (24)	Cohort	1,075	6 × 6 mm	7.57 ± 1.20/ 7.65 ± 0.97	55.4%	Triton DRI-OCT	−0.09 ± 4.34	Mixed (A, AS)	China	30	1,045
Yilmaz Cinar and Ozkan (25)	Cross-sectional	50	3 × 3/6 × 6 mm	12.60 ± 2.83/ 11.64 ± 2.31	38.0%	RTVueXR Avanti (SSADA)	/	Mixed (A, AS)	Turkey	25	25
Araki et al. (15)	Cross-sectional	15	3 × 3 mm	9.8 ± 3.4	73.3%	NIDEK RS-3000, Advance (CODAA)	/	Mixed (A, AS)	Japan	15	/
Chen et al. (16)	Cross-sectional	151	3 × 3 mm	8 ± 0.52	45.7%	RTVue XR (AngioVue version 2017.1.0.155)	4.53 ± 0.39	Mixed (A, AS)	China	85	66
Demirayak et al. (19)	Cross-sectional	38	3 × 3 mm	9.1 ± 2.73	55.3%	RTVue XR (SSADA)	3.34 ± 0.90	Mixed (A, S)	Turkey	17	21
Doguizi et al. (20)	Cross-sectional	97	3 × 3 mm	13.7 ± 3.25	40.2%	RTVue XR (SSADA)	5.31 ± 1.39	A	Turkey	40	57
			6 × 6 mm								
Sobral et al. (23)	Cross-sectional	26	3 × 3 mm	9.2 ± 2.84	53.8%	RTVue XR (SSADA)	3.20 ± 1.85	Mixed (A, S)	Portugal	26	26
Lonngi et al. (22)	Case-control	59	3 × 3 mm	8.0 ± 4.0	56%	RTVue XR (SSADA)	3.86 ± 19.88	Mixed (A, S)	USA	13	50
			6 × 6 mm								

*SD, standard deviation; OCTA, optical coherence tomography angiography; D, diopter; S, strabismic amblyopia; A, anisometropic amblyopia; AS, combined amblyopia patients with strabismus and anisometropia; SSADA, split-spectrum amplitude-decorrelation angiography algorithm; CODAA, complex OCT-signal difference analysis angiography*.

**Table 2:** Evaluation of study quality.

**References**	**Representativeness**	**Selection**	**Ascertainment**	**Adjustment for confounders**	**Assessment of outcome**	**Response rate**	**Scores**	**Quality**
Zhang et al. (26)	☆	☆	☆	☆☆	☆	☆	7	High
Chen et al. (17)	☆	☆	☆	☆	☆	☆	7	High
Cinar et al. (18)	☆	☆	—	☆☆	☆	☆	6	High
Gunzenhauser et al. (21)	☆	☆	☆	☆☆	☆	☆	7	High
Wong et al. (24)	☆	☆	☆	—	☆	☆	5	High
Yilmaz Cinar and Ozkan (25)	☆	☆	☆	☆☆	☆	☆	7	High
Araki et al. (15)	☆	—	☆	☆☆	☆	☆	6	High
Chen et al. (16)	☆	☆	☆	☆☆	☆	☆	7	High
Demirayak et al. (19)	☆	☆	☆	☆☆	☆	☆	7	High
Doguizi et al. (20)	☆	☆	☆	☆☆	☆	☆	7	High
Sobral et al. (23)	☆	☆	☆	☆☆	☆	☆	7	High
Lonngi et al. (22)	☆	☆	☆	☆☆	☆	☆	7	High

*According to the Newcastle-Ottawa scale (NOS), “☆” has different meanings under different item. “☆☆” Represents adjustment for age and other controlled factors*.

### Macular Vessel Density in Amblyopic Eyes Compared With Healthy Control Eyes

**Figure 2:**
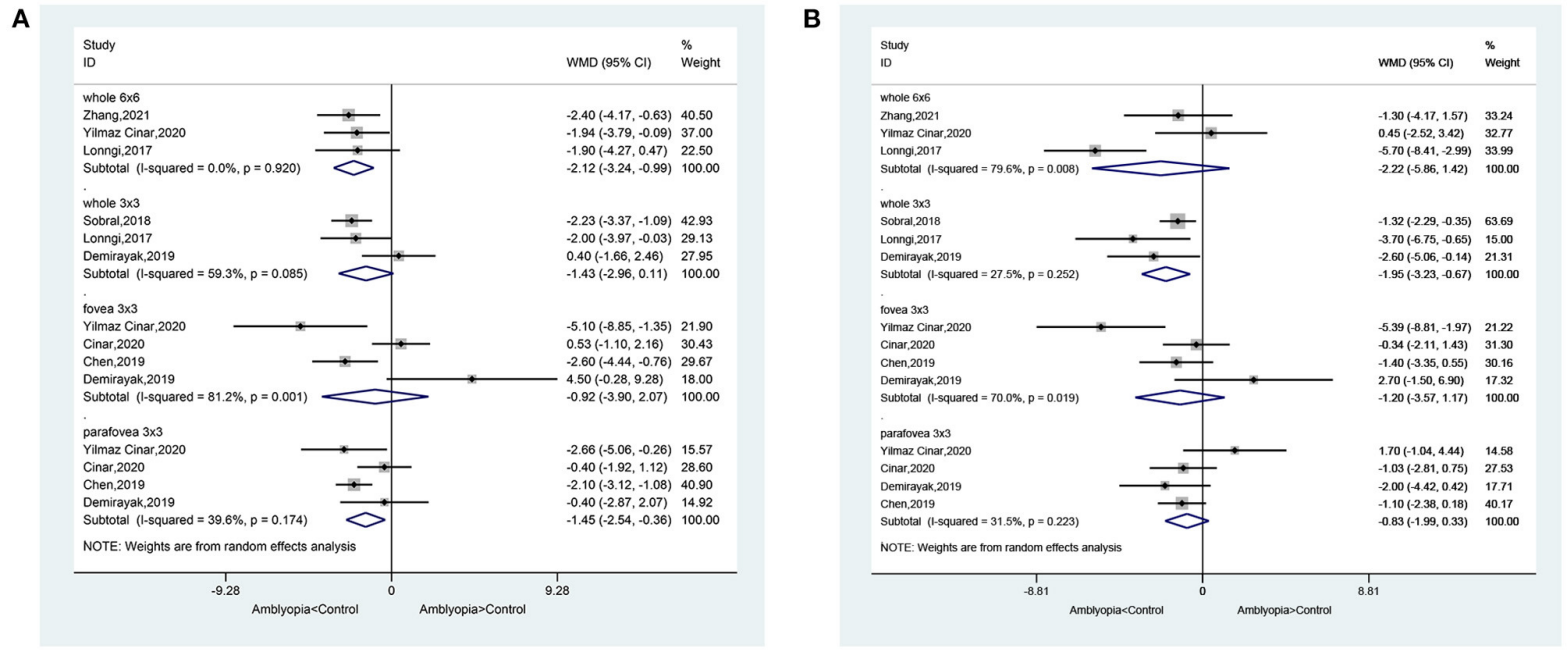
Forest plot of macular vessel density in the superficial capillary plexus (SCP) **(A)** and deep capillary plexus (DCP) **(B)** of amblyopic eyes compared with healthy control eyes.

In the SCP, when considering the whole eye in 6 × 6 volume scan, amblyopia decreased the vessel density (WMD = −2.12, 95%CI: −3.24, −0.99), but not when considering the whole eye in 3 × 3 volume scan (WMD = −1.43, 95%CI: −2.96, 0.11). Heterogeneity was not observed for the 6 × 6 analysis (*I*^2^ = 0.0%, *P* = 0.920), but there was heterogeneity for the 3 × 3 volume scan (*I*^2^ = 59.3%, *P* = 0.085). No impacts were observed when considering the fovea 3 × 3 volume scan (WMD = −0.92, 95%CI: −3.90, 2.07). Heterogeneity was observed (*I*^2^ = 81.2%, *P* = 0.001). When considering the parafovea 3 × 3, the microvessel density in the SCP was reduced in amblyopic eyes (WMD = −1.45, 95%CI: −2.54, −0.36). Heterogeneity was not observed (*I*^2^ = 39.6%, *P* = 0.174) (Figure 2A). In the subgroup analyses, and impact of anisometropia was observed on the whole eye (WMD = −2.40, 95%CI: −4.17, −0.63). Similar effects were observed for mixed amblyopia (WMD = −1.92, 95%CI: −3.38, −0.47; *I*^2^ = 0.0%, *P* = 0.979) (Supplementary Figure 1A). No impact was observed at the fovea level. At the parafovea level, mixed amblyopia affected the microvessel density (WMD = −1.96, 95%CI: −2.84, −1.08; *I*^2^ = 0.0%, *P* = 0.382), but not anisometropia (WMD = −0.40, 95%CI: 1.92, 1.12).

In the DCP, when considering the whole eye 6 × 6, amblyopia did not decrease vessel density (WMD = −2.22, 95%CI: −5.86, 1.42; *I*^2^ = 79.6%, *P* = 0.008), but a difference was observed in the whole eye 3 × 3 (WMD = −1.95, 95%CI: −3.23, −0.67; *I*^2^ = 27.5%, *P* = 0.252). Vessel density was not decreased in the fovea 3 × 3 volume scan (WMD = −1.20, 95%CI: −3.57, 1.17; *I*^2^ = 70.0%, *P* = 0.019). When considering the parafovea, the microvessel density in the DCP was not reduced in amblyopic eyes (WMD = −0.83, 95%CI: −1.99, 0.33; *I*^2^ = 31.5%, *P* = 0.223) (Figure 2B). In the subgroup analyses, there were no impacts of anisometropia or mixed amblyopia on microvessel density compared with healthy eyes (Supplementary Figure 1B).

### Foveal Avascular Zone Area in Amblyopic Eyes Compared With Healthy Control Eyes

**Figure 3:**
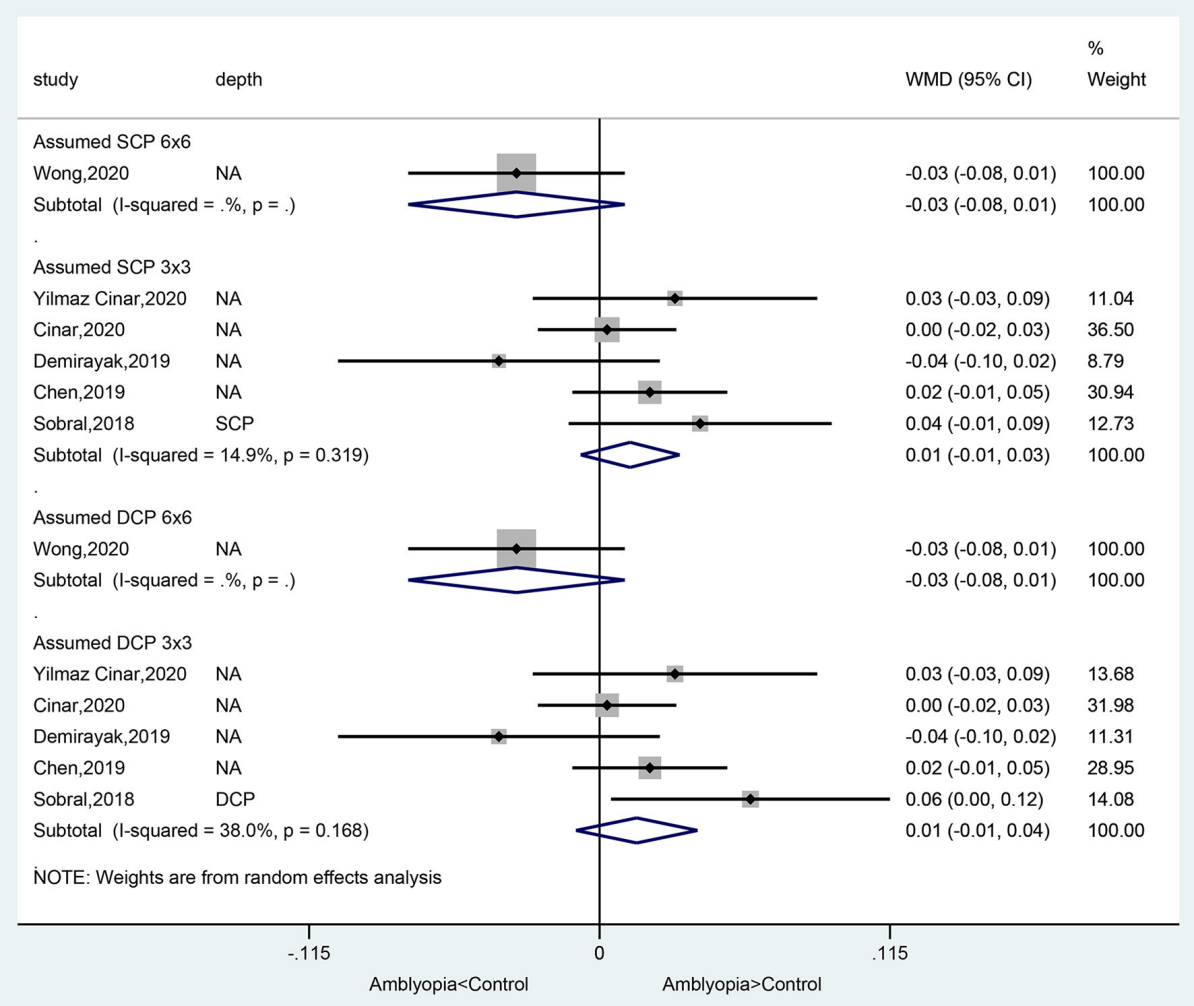
Forest plot of foveal avascular zone area of amblyopic eyes compared with healthy control eyes.

There was no significant difference between amblyopia eyes and healthy control eyes in foveal avascular zone area in the assumed SCP 6 × 6 volume scan (WMD = −0.03, 95%CI: −0.08, 0.01), assumed SCP 3 × 3 volume scan (WMD = 0.01, 95%CI: −0.03, 0.03; *I*^2^ = 14.9%, *P* = 0.319), assumed DCP 6 × 6 volume scan (WMD = −0.03, 95%CI: −0.08, 0.01), and in the assumed DCP 3 × 3 volume scan (WMD = 0.01, 95%CI: −0.01, 0.04; *I*^2^ = 38.0%, *P* = 0.168) (Figure 3).

### Foveal Thickness in Amblyopic Eyes Compared With Healthy Control Eyes

**Figure 4:**
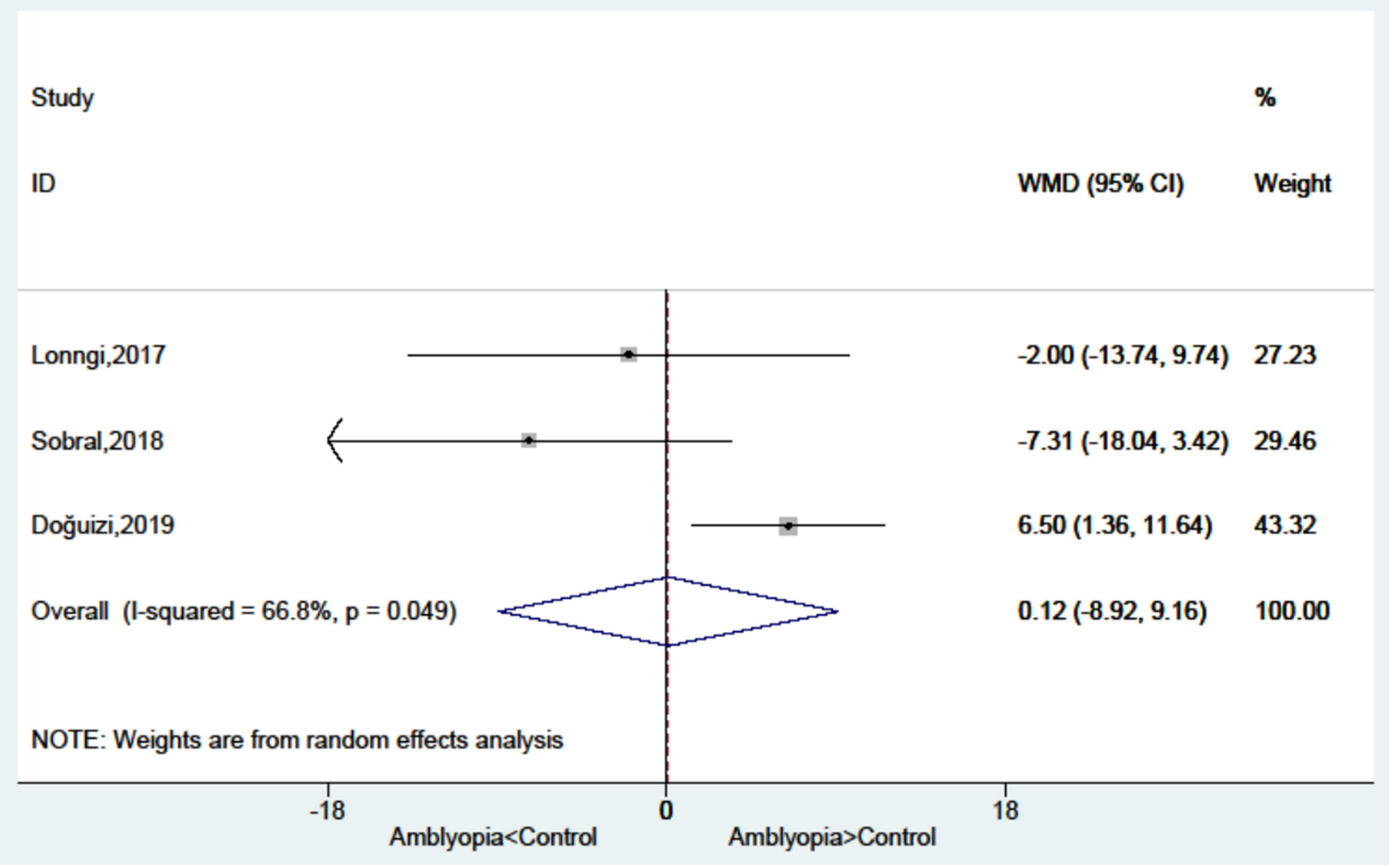
Forest plot of the foveal thickness of amblyopic eyes compared with healthy control eyes.

There was no significant difference between amblyopia eyes and healthy control eyes in foveal thickness (WMD = 0.12, 95%CI: −8.92, 9.16; *I*^2^ = 66.8%, *P* = 0.049) (Figure 4).

### Macular Vessel Density in Amblyopic Eyes Compared With Fellow Eyes

**Figure 5:**
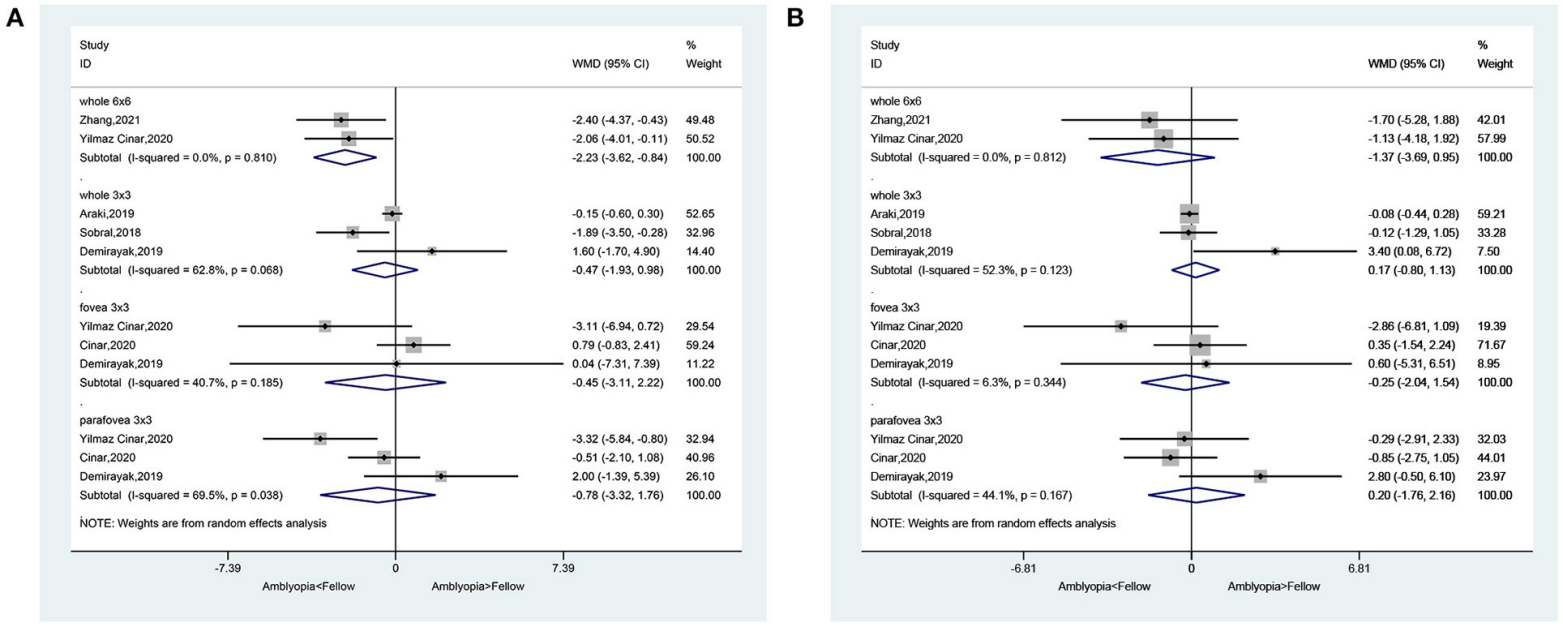
Forest plot of macular vessel density in the superficial capillary plexus (SCP) **(A)** and deep capillary plexus (DCP) **(B)** of amblyopic eyes compared with fellow eyes.

In the SCP, amblyopia decrease the vessel density when considering the whole eye in 6 × 6 volume scan (WMD = −2.23, 95%CI: −3.62, −0.84; *I*^2^ = 0.0%, *P* = 0.810), but not for the whole eye in 3 × 3 volume scan (WMD = −0.47, 95%CI: −1.93, 0.98; *I*^2^ = 62.8%, *P* = 0.068), fovea 3 × 3 (WMD = −0.45, 95%CI: −3.11, 2.22; *I*^2^ = 40.7%, *P* = 0.185), and parafovea (WMD = −0.78, 95%CI: −3.32, 1.76; *I*^2^ = 69.5%, *P* = 0.038) (Figure 5A). No differences were observed in the subgroup analyses of anisometropia and mixed amblyopia (Supplementary Figure 1C).

In the DCP, amblyopia did not decrease the vessel density when considering the whole eye in 6 × 6 volume scan (WMD = −1.37, 95%CI: −3.69, 0.95, *I*^2^ = 0.0%, *P* = 0.812), whole eye 3 × 3 (WMD = 0.17, 95%CI: −0.80, 1.13; *I*^2^ = 52.3%, *P* = 0.123), fovea 3 × 3 volume scan (WMD = −0.25, 95%CI: −2.04, 1.54; *I*^2^ = 6.3%, *P* = 0.344), and parafovea 3 × 3 volume scan (WMD = 0.20, 95%CI: −1.76, 2.16; *I*^2^ = 44.1%, *P* = 0.167) (Figure 5B). No differences were observed in the subgroup analyses of anisometropia and mixed amblyopia (Supplementary Figure 1D).

### Foveal Avascular Zone Area in Amblyopic Eyes Compared With Fellow Eyes

**Figure 6:**
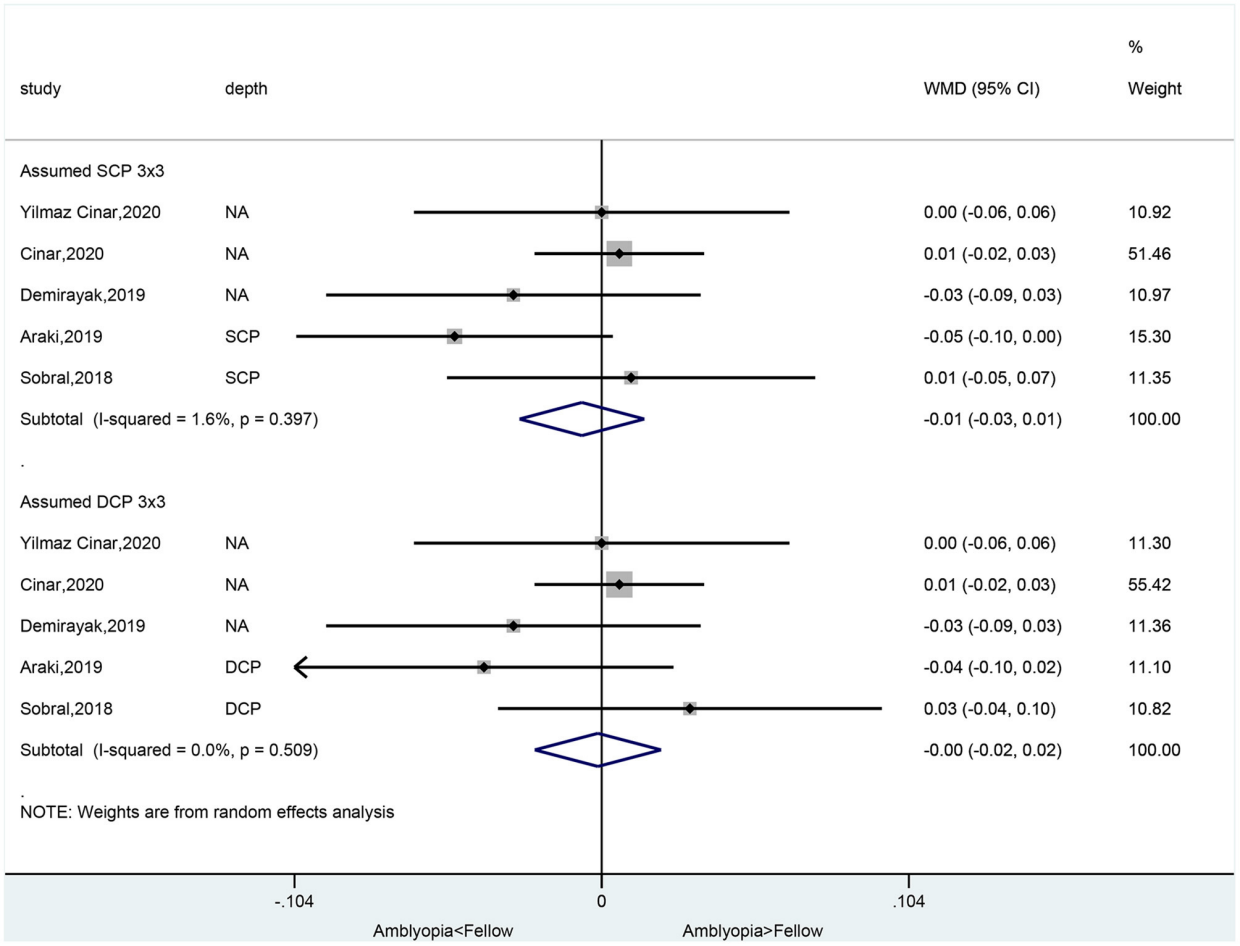
Forest plot of foveal avascular zone area of amblyopic eyes compared with fellow eyes.

There was no significant difference between amblyopia eyes and fellow eyes in foveal avascular zone area in the assumed SCP 3 × 3 volume scan (WMD = −0.01, 95%CI: −0.03, 0.01; *I*^2^ = 1.6%, *P* = 0.397) and in the assumed DCP 3 × 3 volume scan (WMD = −0.00, 95%CI: −0.02, 0.02; *I*^2^ = 0.0%, *P* = 0.509) (Figure 6).

### Foveal Thickness in Amblyopic Eyes Compared With Fellow Eyes

**Figure 7:**
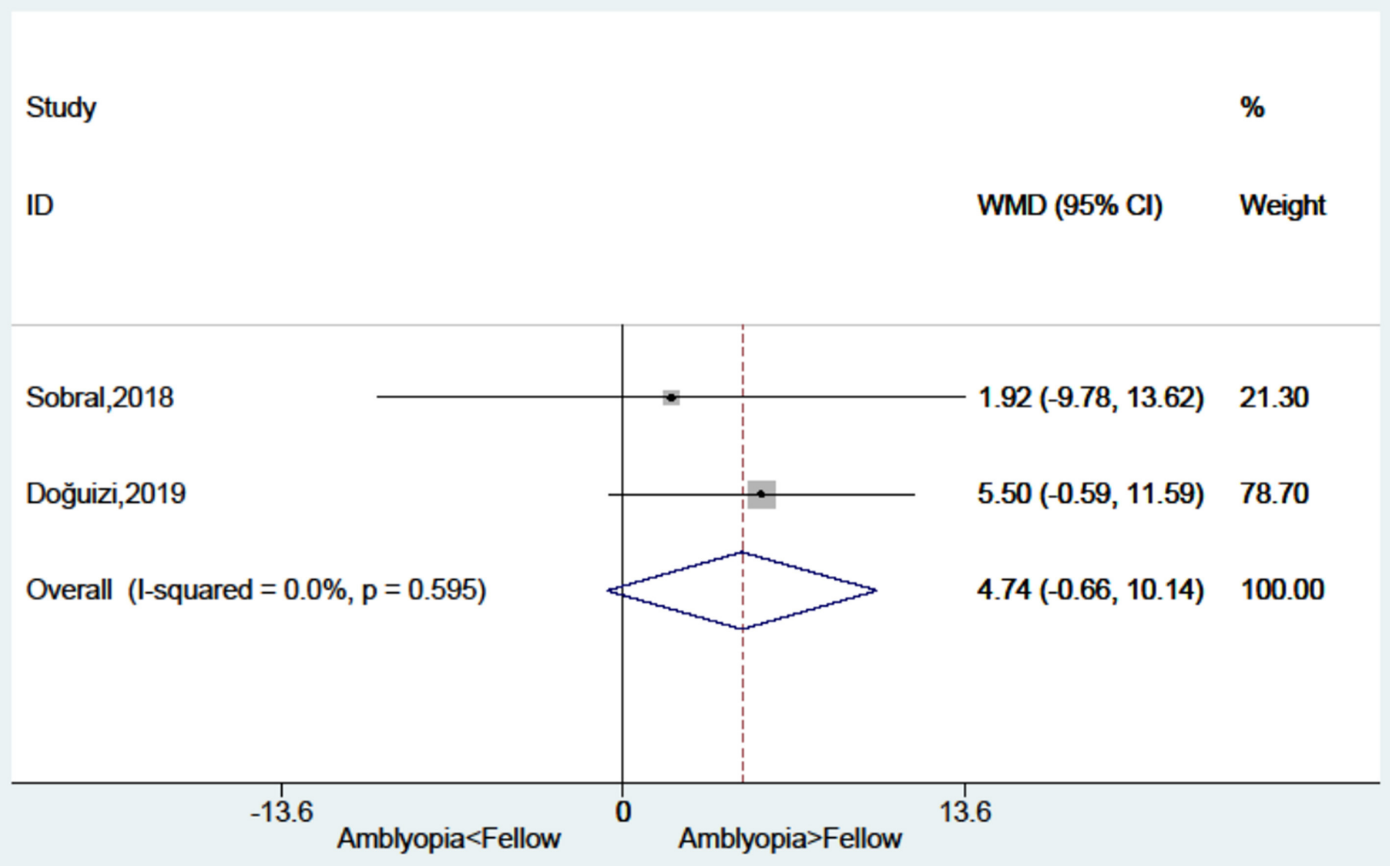
Forest plot of the foveal thickness of amblyopic eyes compared with fellow eyes.

There was no significant difference between amblyopia eyes and fellow eyes in foveal thickness (WMD = 4.74, 95%CI: −0.66, 10.14; *I*^2^ = 0.0%, *P* = 0.595) (Figure 7).

### Changes in Macular Vessel Density in SCP and DCP Before and After Treatments

**Figure 8:**
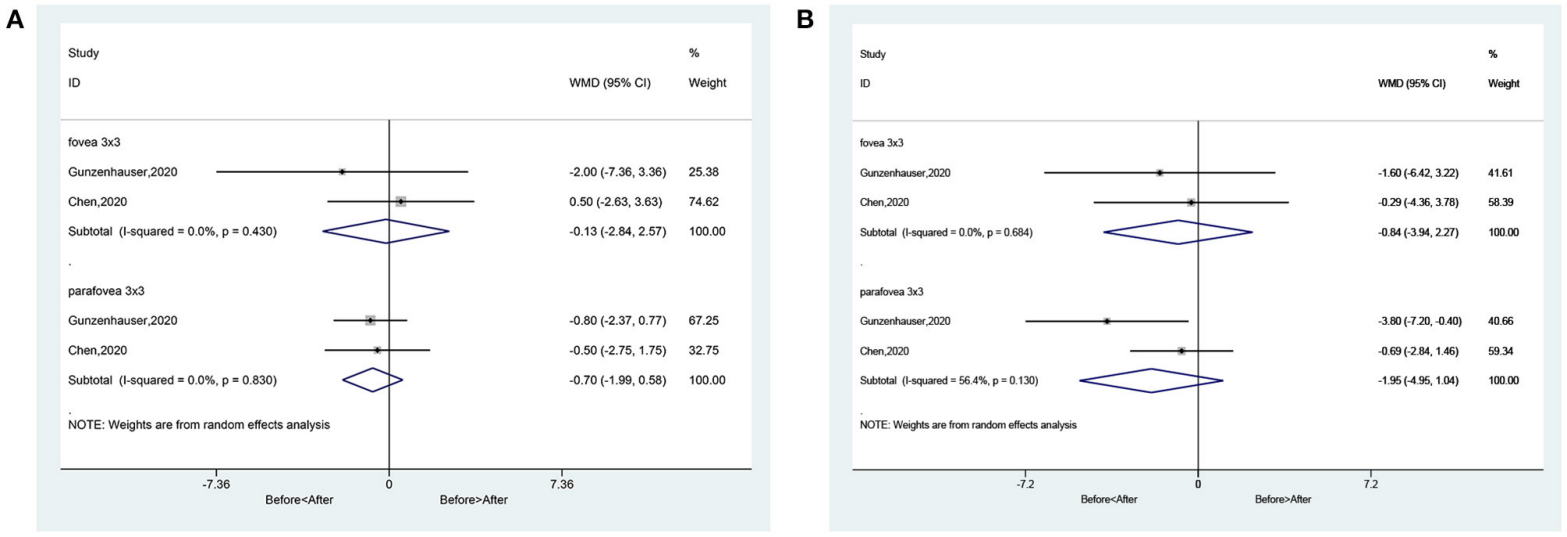
Forest plot of macular vessel density in the superficial capillary plexus (SCP) **(A)** and deep capillary plexus (DCP) **(B)** of difference between pre-treatment and post-treatment in amblyopic eyes.

There were no changes in fovea 3 × 3 volume scan (WMD = −0.13, 95%CI: −2.84, 2.57; *I*^2^ = 0.0%, *P* = 0.430) and parafovea 3 × 3 volume scan (WMD = −0.70, 95%CI: −1.99, 0.58; *I*^2^ = 0.0%, *P* = 0.830) in the SCP (Figure 8A). There were no changes in fovea 3 × 3 volume scan (WMD = −0.84, 95%CI: −3.94, 2.27; *I*^2^ = 0.0%, *P* = 0.684) and parafovea 3 × 3 volume scan (WMD = −1.95, 95%CI: −4.95, 1.04; *I*^2^ = 56.4%, *P* = 0.130) in the DCP (Figure 8B).

### Publication Bias

No significant publication bias was observed (Supplementary Figure 2).

## Discussion

OCTA is a novel rapid and non-invasive method for the angiography of the retina and choroid (9, 10), but the studies yield conflicting results. Therefore, the aim of this meta-analysis was to examine the quantitative measurements of OCTA in children with amblyopia. The results indicate that according to OCTA, amblyopic eyes had lower vessel density in parafoveal SCP and DCP compared with healthy control eyes, but not compared with fellow eyes. There were no significant differences regarding the foveal avascular zone area and foveal thickness between amblyopic and non-amblyopic eyes.

Because of the small number of patients included in the previous studies, the present meta-analysis increased the power to provide a more convincing assessment of OCTA in amblyopia. Among the parameters measured by OCTA, the parafoveal vessel density was different between amblyopic and healthy control eyes, and this difference was not observed when compared with the fellow eye. This suggests that the pathogenesis of amblyopia involves a reduced vascularization of the affected eye but that this process could also be observed in the fellow normal eye. This was observed by Sobral et al. (23). Chatzistefanou et al. (27) and Meier and Giaschi (28) also observed that unilateral amblyopia affected the fellow eye and that the fellow eye cannot be considered equivalent to a normal healthy eye. Of note, different results were obtained when considering the whole eye in 6 × 6 and 3 × 3 volume scan, suggesting that different measurement methodologies affected the results.

In the present meta-analysis, no difference was observed at the fovea level between amblyopic eyes and normal healthy or fellow eyes. This is in contradiction with a meta-analysis of OCT that showed thicker fovea in amblyopic eyes compared with control eyes (7). This discrepancy could be due to the differences in the methods of measurement and the included studies.

Nevertheless, there is literature supporting abnormal eye development in amblyopia. Indeed, rapid visual development is observed in the sensitive visual development period, which ends by 6–7 years of age (29). During this critical period, any obstacles to the normal visual environment increase the risk of amblyopia, leading to changes in cortical neurons and their synapses (29). The blurred vision will lead to insufficient stimulation in the amblyopic eye, which affects the maturation of the macula (30), which could contribute to decreased need for oxygen supply and decreased vascularization. The lack of normal vision could also lead to reduced apoptosis of the ganglion cells in the amblyopic eyes, leading to the thicker fovea (6), as observed by Li et al. (7), but this was not observed in the present study.

Of course, the results of the present meta-analysis must be considered in relation to the study limitations. First, unpublished studies, conference abstracts, and non-English studies were not included. In addition, because of the small number of studies, bias might still be present. Second, because of the small number of studies, the influence of treatments could not be assessed. Such treatments have been shown to influence foveal thickness in amblyopia (31), and only two of the included studies reported treatments. Miki et al. (32) and Tugcu et al. (33) reported differences in foveal thickness between different types of amblyopia, but this could not be examined here. Furthermore, all included studies were cross-sectional and case-control studies, and the cause-to-effect relationship of amblyopia and reduced vascularization could not be examined. Third, the correlations between the severity of the amblyopia, the types of amblyopia, and the microvessel density of the amblyopic eyes could not be investigated because such data were not reported in the included studies. We performed subgroup analyses for the types of amblyopia, but the numbers of studies in some subgroups were small. Nevertheless, OCTA is a recent technology, the area is still being explored, and the number of studies is small. Future studies should provide additional data, especially clinical trials.

In conclusion, according to OCTA, amblyopic eyes had lower vessel density in parafoveal SCP and DCP compared with healthy control eyes, but not compared with fellow eyes. There were no significant differences regarding the foveal avascular zone area and foveal thickness between amblyopic and non-amblyopic eyes.

## Data Availability Statement

The original contributions presented in the study are included in the article/Supplementary Material, further inquiries can be directed to the corresponding author.

## Author Contributions

LG, YG, and XS: substantially contributed to conception or design. LG and PZ: contributed to acquisition and analysis of data. LG: drafted the manuscript for important content and gave final approval. YG, FH, PZ, and XS: critically revised the manuscript for important intellectual content. All authors contributed to the article and approved the submitted version.

## Conflict of Interest

The authors declare that the research was conducted in the absence of any commercial or financial relationships that could be construed as a potential conflict of interest.

## References

[1] Gunton KB. Advances in amblyopia: what have we learned from PEDIG trials? Pediatrics. (2013) 131:540–7. 10.1542/peds.2012-1622 23382445

[2] DeSantis D. Amblyopia. Pediatr Clin North Am. (2014) 61:505–18. 10.1016/j.pcl.2014.03.006 24852148

[3] Pescosolido N Stefanucci A Buomprisco G Fazio S. Amblyopia treatment strategies and new drug therapies. J Pediatr Ophthalmol Strabismus. (2014) 51:78–86. 10.3928/01913913-20130107-01 24410693

[4] Taylor K Elliott S. Interventions for strabismic amblyopia. Cochrane Database Syst Rev. (2014) 7:CD006461. 10.1002/14651858.CD006461.pub4 PMC10816941 25051925

[5] Potts RA Dreher B Bennett MR. The loss of ganglion cells in the developing retina of the rat. Brain Res. (1982) 255:481–6. 10.1016/0165-3806(82)90013-x 7066701

[6] Yen MY Cheng CY Wang AG. Retinal nerve fiber layer thickness in unilateral amblyopia. Invest Ophthalmol Vis Sci. (2004) 45:2224–30. 10.1167/iovs.03-0297 15223799

[7] Li J Ji P Yu M. Meta-analysis of retinal changes in unilateral amblyopia using optical coherence tomography. Eur J Ophthalmol. (2015) 25:400–9. 10.5301/ejo.5000583 25837638

[8] Fingler J Readhead C Schwartz DM Fraser SE. Phase-contrast OCT imaging of transverse flows in the mouse retina and choroid. Invest Ophthalmol Vis Sci. (2008) 49:5055–9. 10.1167/iovs.07-1627 18566457

[9] de Carlo TE Romano A Waheed NK Duker JS. A review of optical coherence tomography angiography (OCTA). Int J Retina Vitreous. (2015) 1:5. 10.1186/s40942-015-0005-8 27847598 PMC5066513

[10] Gao SS Jia Y Zhang M Su JP Liu G Hwang TS. Optical coherence tomography angiography. Invest Ophthalmol Vis Sci. (2016) 57:OCT27–36. 10.1167/iovs.15-19043 27409483 PMC4968919

[11] Jia Y Morrison JC Tokayer J Tan O Lombardi L Baumann B. Quantitative OCT angiography of optic nerve head blood flow. Biomed Opt Express. (2012) 3:3127–37. 10.1364/BOE.3.003127 23243564 PMC3521313

[12] Kaizu Y Nakao S Arima M Hayami T Wada I Yamaguchi M. Flow density in optical coherence tomography angiography is useful for retinopathy diagnosis in diabetic patients. Sci Rep. (2019) 9:8668. 10.1038/s41598-019-45194-z 31209251 PMC6572797

[13] Lo CK Mertz D Loeb M. Newcastle-Ottawa Scale: comparing reviewers' to authors' assessments. BMC Med Res Methodol. (2014) 14:45. 10.1186/1471-2288-14-45 24690082 PMC4021422

[14] Yeung SSY Reijnierse EM Pham VK Trappenburg MC Lim WK Meskers CGM. Sarcopenia and its association with falls and fractures in older adults: a systematic review and meta-analysis. J Cachexia Sarcopenia Muscle. (2019) 10:485–500. 10.1002/jcsm.12411 30993881 PMC6596401

[15] Araki S Miki A Goto K Yamashita T Yoneda T Haruishi K. Foveal avascular zone and macular vessel density after correction for magnification error in unilateral amblyopia using optical coherence tomography angiography. BMC Ophthalmol. (2019) 19:171. 10.1186/s12886-019-1177-z 31382925 PMC6683430

[16] Chen W Lou J Thorn F Wang Y Mao J Wang Q. Retinal microvasculature in amblyopic children and the quantitative relationship between retinal perfusion and thickness. Invest Ophthalmol Vis Sci. (2019) 60:1185–91. 10.1167/iovs.18-26416 30913291

[17] Chen W Thorn F Deng R Li X Lou J Wang Y. Macular microvasculature density changes in anisometropic amblyopic eyes after successful treatment. J Ophthalmol. (2020) 2020:8879175. 10.1155/2020/8879175

[18] Cinar E Yuce B Aslan F Erbakan G. Comparison of retinal vascular structure in eyes with and without amblyopia by optical coherence tomography angiography. J Pediatr Ophthalmol Strabismus. (2020) 57:48–53. 10.3928/01913913-20191004-01 31972041

[19] Demirayak B Vural A Onur IU Kaya FS Yigit FU. Analysis of macular vessel density and foveal avascular zone using spectral-domain optical coherence tomography angiography in children with amblyopia. J Pediatr Ophthalmol Strabismus. (2019) 56:55–9. 10.3928/01913913-20181003-02 30371915

[20] Doguizi S Yilmazoglu M Kiziltoprak H Sekeroglu MA Yilmazbaş P. Quantitative analysis of retinal microcirculation in children with hyperopic anisometropic amblyopia: an optical coherence tomography angiography study. J AAPOS. (2019) 23:201.e1–e5. 10.1016/j.jaapos.2019.01.017 31112776

[21] Gunzenhauser RC Tsui I Velez FG Fung SS Demer JL Suh SY. Comparison of pre-treatment vs. post-treatment retinal vessel density in children with amblyopia. J Binocul Vis Ocul Motil. (2020) 70:79–85. 10.1080/2576117x.2020.1760695 32412887

[22] Lonngi M Velez FG Tsui I Davila JP Rahimi M Chan C. Spectral-domain optical coherence tomographic angiography in children with amblyopia. JAMA Ophthalmol. (2017) 135:1086–91. 10.1001/jamaophthalmol.2017.3423 28910439 PMC5710487

[23] Sobral I Rodrigues TM Soares M Seara M Monteiro M Paiva C. OCT angiography findings in children with amblyopia. J AAPOS. (2018) 22:286–9.e2. 10.1016/j.jaapos.2018.03.009 30031875

[24] Wong ES Zhang XJ Yuan N Li J Pang CP Chen L. Association of optical coherence tomography angiography metrics with detection of impaired macular microvasculature and decreased vision in amblyopic eyes: the Hong Kong children eye study. JAMA Ophthalmol. (2020) 138:858–65. 10.1001/jamaophthalmol.2020.2220 32584368 PMC7317658

[25] Yilmaz Cinar FG Ozkan G. Macular capillary system and ganglion cell-layer complex of the amblyopic eye with optical cohorence tomography angiography and optical cohorence tomography. Int Ophthalmol. (2020) 41:675–86. 10.1007/s10792-020-01624-w 33079311

[26] Zhang T Xie S Liu Y Xue C Zhang W. Effect of amblyopia treatment on macular microvasculature in children with anisometropic amblyopia using optical coherence tomographic angiography. Sci Rep. (2021) 11:39. 10.1038/s41598-020-79585-4 33420155 PMC7794286

[27] Chatzistefanou KI Theodossiadis GP Damanakis AG Ladas ID Moschos MN Chimonidou E. Contrast sensitivity in amblyopia: the fellow eye of untreated and successfully treated amblyopes. J AAPOS. (2005) 9:468–74. 10.1016/j.jaapos.2005.05.002 16213398

[28] Meier K Giaschi D. Unilateral amblyopia affects two eyes: fellow eye deficits in amblyopia. Invest Ophthalmol Vis Sci. (2017) 58:1779–800. 10.1167/iovs.16-20964 28346616

[29] Lewis TL Maurer D. Multiple sensitive periods in human visual development: evidence from visually deprived children. Dev Psychobiol. (2005) 46:163–83. 10.1002/dev.20055 15772974

[30] Wu SQ Zhu LW Xu QB Xu JL Zhang Y. Macular and peripapillary retinal nerve fiber layer thickness in children with hyperopic anisometropic amblyopia. Int J Ophthalmol. (2013) 6:85–9. 10.3980/j.issn.2222-3959.2013.01.18 23550031 PMC3580257

[31] Huynh SC Samarawickrama C Wang XY Rochtchina E Wong TY Gole GA. Macular and nerve fiber layer thickness in amblyopia: the Sydney childhood eye study. Ophthalmology. (2009) 116:1604–9. 10.1016/j.ophtha.2009.03.013 19560205

[32] Miki A Shirakashi M Yaoeda K Kabasawa Y Ueki S Takagi M. Retinal nerve fiber layer thickness in recovered and persistent amblyopia. Clin Ophthalmol. (2010) 4:1061–4. 10.2147/opth.s13145 20922043 PMC2946998

[33] Tugcu B Araz-Ersan B Erdogan ET Tarakcioglu H Coskun C Yigit U. Structural and functional comparison of the persistent and resolved amblyopia. Doc Ophthalmol. (2014) 128:101–9. 10.1007/s10633-013-9422-x 24343574

