# Supplementary material for: Assessment of Foveal Avascular Zone and Macular Vascular Plexus Density in Children With Unilateral Amblyopia: A Systemic Review and Meta-Analysis
Source: Front Pediatr. 2021 May 21;9:620565. doi: 10.3389/fped.2021.620565 (PMC8175854; doi:10.3389/fped.2021.620565)
Supplement: Supplementary file 1 [file Data_Sheet_1.doc]

**
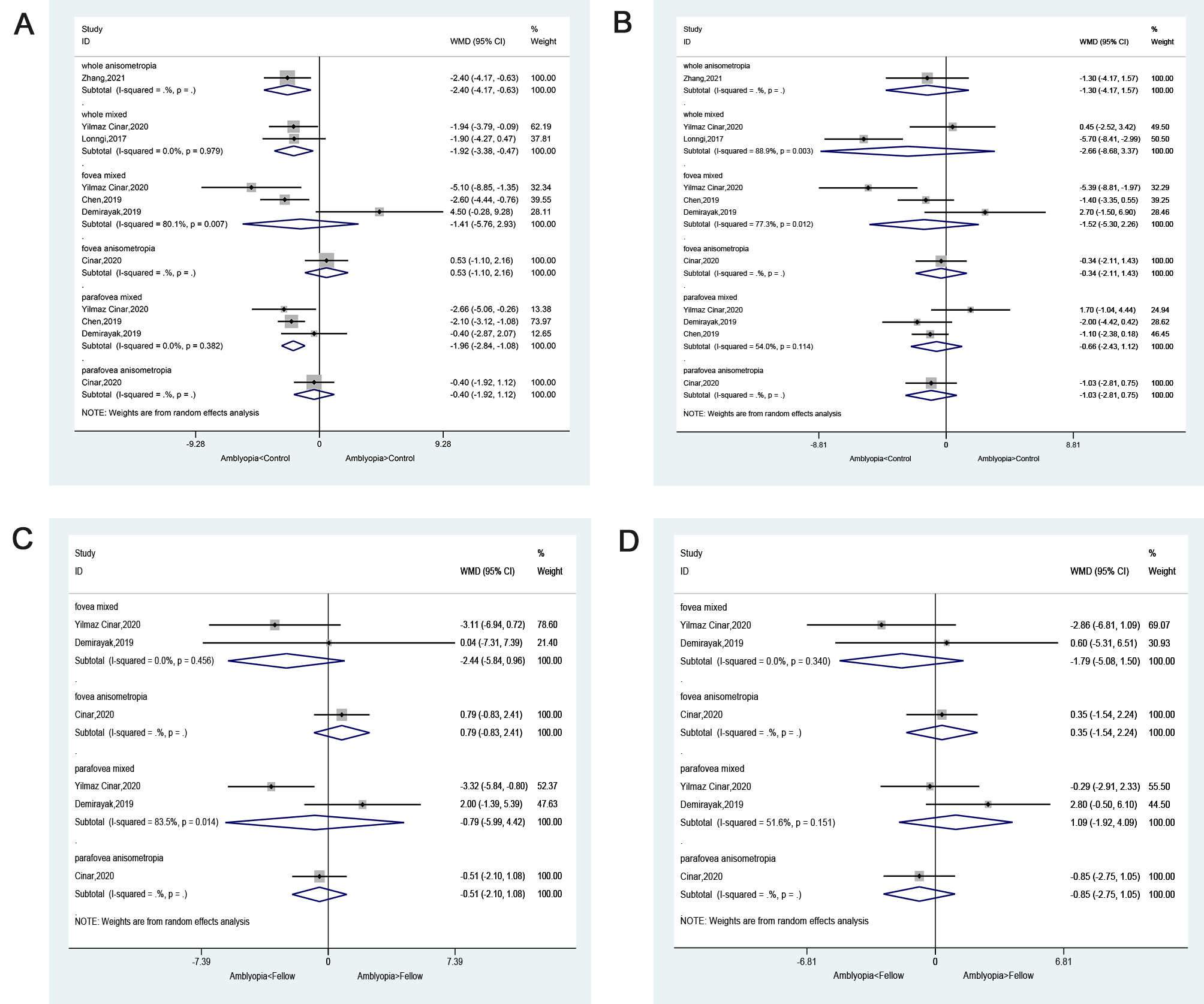
**

**Supplementary Figure S1.** Subgroup analysis according to the types of amblyopia. Forest plot of macular vessel density in the superficial capillary plexus (SCP) (A) and deep capillary plexus (DCP) (B) of amblyopic eyes compared with healthy control eyes. Forest plot of macular vessel density in the superficial capillary plexus (SCP) (C) and deep capillary plexus (DCP) (D) of amblyopic eyes compared with fellow eyes.


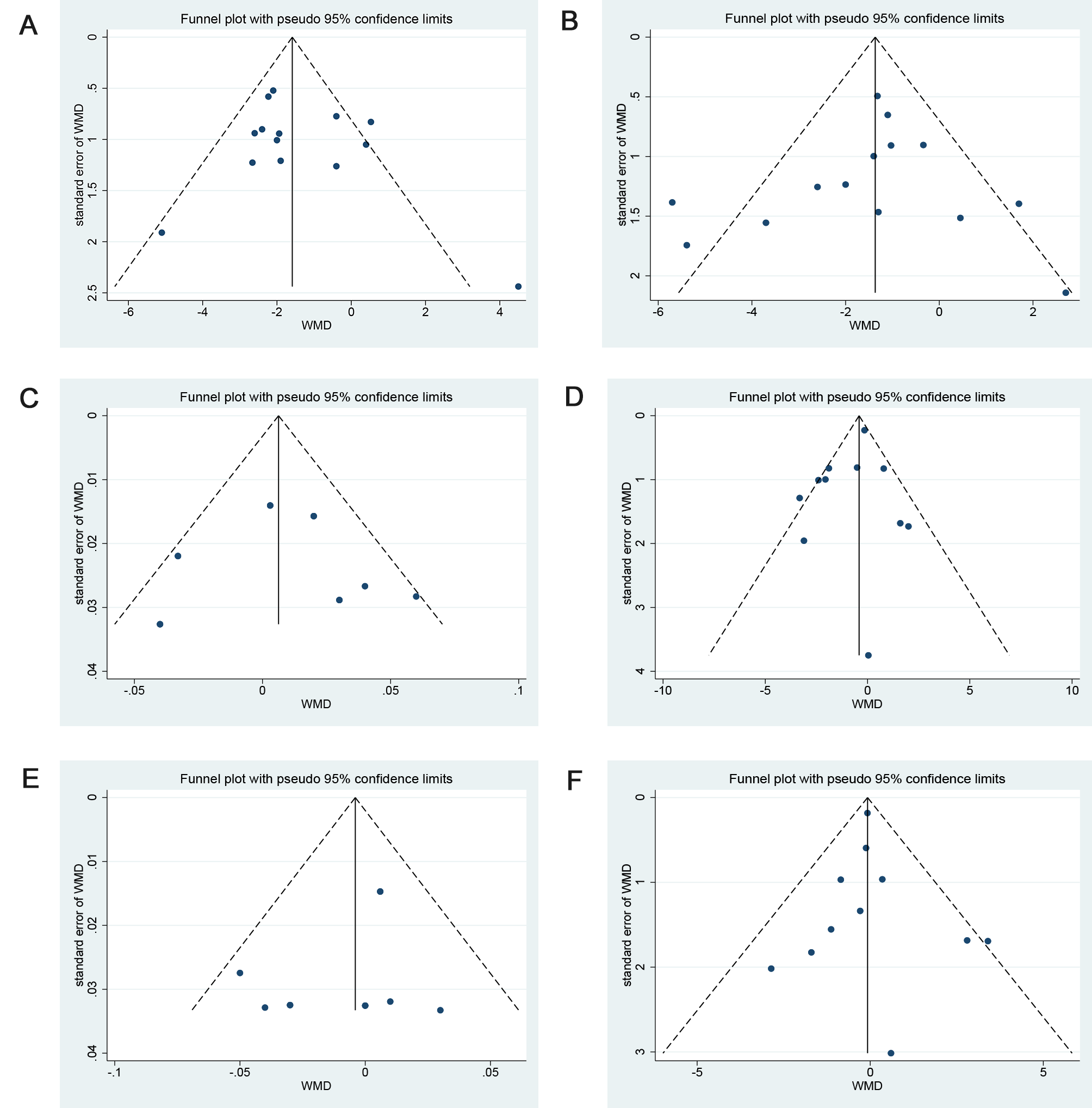


**Supplementary Figure S2.** Funnel plot of macular vessel density in the superficial capillary plexus (SCP) (A) and deep capillary plexus (DCP) (B) of amblyopic eyes compared with healthy control eyes, foveal avascular zone area of amblyopic eyes compared with healthy control eyes (C) and macular vessel density in the superficial capillary plexus (SCP) (D) and deep capillary plexus (DCP) (E) of amblyopic eyes compared with fellow eyes, and foveal avascular zone area of amblyopic eyes compared with fellow eyes (F).
